# Regulatory role of E-NTPase/E-NTPDase in Ca^2+^/Mg^2+ ^transport via gated channel

**DOI:** 10.1186/1742-4682-1-3

**Published:** 2004-08-12

**Authors:** Hans M Schreiber, Subburaj Kannan

**Affiliations:** 1 Division of Gastroenterology, School of Medicine, University of Pennsylvania, Philadelphia, PA 19104, USA; 2 Departments of Microbiology and Immunology, School of Medicine, PO Box 25056, University of Texas Medical Branch, 300 University Boulevard, Galveston, Texas, 77550 USA

## Abstract

**Background:**

E-NTPase/E-NTPDase is activated by millimolar concentrations of Ca^2+ ^or Mg^2+ ^with a pH optimum of 7.5 for the hydrolysis of extracellular NTP and NDP. It has been generally accepted that E-NTPase/E-NTPDase plays regulatory role in purinergic signalling, but other functions may yet be discovered.

**Results:**

In this article it is proposed on the basis of published data that E-NTPase/E-NTPDase could play a role in the influx and efflux of Ca^2+^and Mg^2+ ^in vivo.

**Conclusions:**

Attenuation of extracellular Ca2+ influx by rat cardiac sarcoplasmic anti-E-NTPase antibodies and oligomerization studies on mammalian CD39 conclusively point towards the existence of a new channel in the membrane. Further studies on these properties of the E-NTPase/E-NTPDase may provide detailed mechanisms and identify the potential patho-physiological significance.

## Background

The mechanism by which [Ca^2+^]_i _is increased in excitable cells differs from that obtaining in non-excitable cells. Excitable cells exhibit an action potential, a substantial general depolarization of the plasma membrane, in response to depolarizing stimuli; influx of Ca^2+ ^occurs via plasma membrane Ca^2+ ^channels and/or release from sarco (endo) plasmic reticulum via ryanodine-receptor Ca^2+ ^channels which regulate the excitation – contraction coupling [1,2]. The factors that determine the extent of Ca^2+ ^entry are **(i) **magnitude of the membrane potential and (**ii) **magnitude of the transmembrane Ca^2+ ^gradient. These two factors also determine whether Ca^2+ ^or Mg^2+ ^enters and the time (probably milliseconds) that elapses between channel opening and termination of Ca^2+ ^or Mg^2+ ^transport [3].

In non-excitable cells, the increase in [Ca^2+^]_i _results from influx of Ca^2+ ^across the plasma membrane and Ca^2+ ^release from the endoplasmic reticulum. Ca^2+ ^release from the SER depends on the binding of inositol 1,4,5-triphosphate (InsP_3_) to its receptor Ca^2+^channels, and also on Ca^2+ ^binding to ryanodine receptor – Ca^2+^channels.

Ca^2+ ^is removed from the cell by the following means. **i: **the sarco (endo) plasmic reticular Ca^2+ ^pump ATPase (SERCA), which transports Ca^2+ ^from the cytoplasm into the SER lumen (~70% of the activator Ca^2+^); **ii: **The plasma membrane Ca^2+ ^pump ATPase (PMCA), which exports Ca^2+ ^across the plasma membrane (~1% of the activator Ca^2+^); **iii: **Mitochondrial Ca^2+^Uniporters (mCa^2+ ^uniporters), which transport Ca^2+ ^into mitochondria (~1% of the activator Ca^2+^);**iv: **the Na^+^/Ca^2+ ^exchanger (28% of the activator Ca^2+^). This last transport system is reversible but under normal physiological conditions, in the Ca^2+ ^extrusion mode, it exhibits a stoichiometry of 3 Na^+^influx/1 Ca^2+ ^efflux [4].

Ca^2+ ^enters animal cells via **(i) **voltage-operated Ca^2+^channels **(VOCC), **(**ii) **ligand gated non-specific cation channels **(LGCCS), **and (**iii) **stretch/receptor activated non-specific Ca^2+ ^channels **(RACC) **[4,5]. A "receptor operated Ca^2+ ^channel" **(ROCC) **is defined as a plasma membrane Ca^2+ ^channel other than VOCC or RACC. VOCC opening depends on membrane depolarization, whereas RACC opening depends on both direct and indirect activation of membrane bound receptors. In contrast, ROCC opening depends solely on agonist-receptor interaction. It has also been suggested that mobile intracellular messengers such as elevated [Ca2+]i play a role in ROCC opening [5,6]. Different types of ROCC are activated (opened) by diverse cell signaling mechanisms such as ligand specificity, increase in [Ca^2+^]_I_, increase in [cAMP]_i _[7] and activation/inactivation of specific trimeric G proteins [8].

Opening of Ca^2+ ^channels must be a highly regulated event involving physical movement of channel components inclusive of the alteration in channel protein conformation; Also, an extracellular source of free energy (ΔG) could be of critical importance. This might be supplied by E-NTPase/E-NTPDase mediated hydrolysis of NTP/NDP. Co-ordination of this process might play a role in the opening of Ca^2+ ^channels, independently of membrane depolarization or other factors.

The biochemical, structural, and functional properties of E-type nucleotidases have been covered in several excellent reviews: **i**. Extracellular metabolism [9]; **ii**. purine signalling [10,11]; **iii**. adhesion [12]; **iv**. transporter functions [13]; **v**. pathophysiology [14,15].

### Rationale for the proposed hypothesis: E-NTPase/E-NTPDase mediated Ca^2+^/Mg^2+ ^transport

It has been suggested that Ca^2+ ^entry during the slow inward current in normal myocardium involves membrane-bound channels potentially controlled and/or regulated by metabolic energy transfer from unknown sources, though Ca^2+ ^enters the cell down its concentration gradient [16]. Electrical stimulation and membrane phosphorylation by cAMP-dependent protein kinase have been shown to increase E-NTPase/E-NTPDase activity. Metal ions such as Mn^2+^, Co^2+^, Ni^2+ ^and La^2+ ^that attenuate Ca^2+ ^influx also inhibit the E-NTPase. In the late stages of heart failure the E-NTPase is down regulated. Activation of E-NTPase by various concentrations of Ca^2+ ^has been shown to correlate linearly with cardiac contractile force development [17].

**"Calcium paradox" **is defined as irreversible functional and structural protein loss in the isolated heart that is first perfused with Ca^2+^-free buffer and then reperfused with Ca^2+^-containing buffer [18]. E-NTPase activity is highest during the initial phases of reperfusion, which might favour the initial Ca^2+ ^influx that causes Ca^2+ ^overload. During the later stages of reperfusion with Ca^2+^-containing buffer there is a loss of E-NTPase activity. During mild stages of Ca^2+ ^paradox, E-NTPase retains its function and continues to favour Ca^2+ ^influx, resulting in the development of intracellular Ca^2+ ^overloads. However, during severe stages of calcium paradox, impaired E-NTPase activity may contribute to irreversible failure of contractile force recovery [19].

To date there is no report describing the detailed mechanism of E-NTPase/E-NTPDase-mediated channel gating and its role in Ca^2+^/Mg^2+ ^transport. In this article an attempt is made to delineate the molecular mechanism of Ca^2+^/Mg^2+ ^transport, identifying the source of energy and the activation and termination of the process. The central issues are:

**a**. How the metabolic energy from nucleotide hydrolysis is effectively utilized in channel opening;

**b**. What stage of the opening/closing cycle requires energy;

**c**. By what (probable) mechanism the proposed scheme is completed;

**d**. How, if at all, homeostasis is affected

The current hypothetical proposal is set out in three sections with appropriate illustrations.

### Phase I: Activation

identifies the evidence that leads to the current proposal and describes how the metabolic energy from nucleotide triphosphate hydrolysis is utilised to assemble a functional homo-oligomer of the E-NTPase/E-NTPDase, forming a channel that is subsequently opened.

### Phase II: Suggested: Ca^2+^/Mg^2+^Transport

Describes, with supporting evidence, how the energy released from [NTP] o/ [NDP] o hydrolysis might be utilized for opening the channel formed by the homo-oligomeric ENTPase/E-NTPDase.

### Phase III: Termination of the transport processes

outlines the intracellular and extracellular factors that would influence the termination of the Ca^2+^/Mg^2+ ^transport processes, and the experimental evidence obtained in favor of the whole proposal.

### Phase I: Activation of E-NTPase/E-NTPDase and channel formation

**Figure 1:**
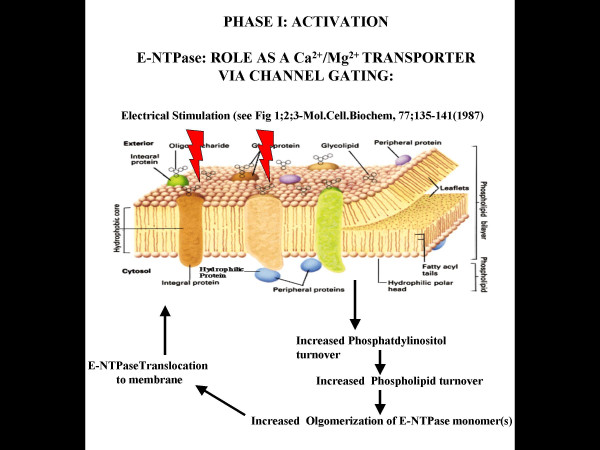
**Phase I: Activation. **Based on direct experimental evidence, suppose that in response to electrical stimuli, an increased phosphatidylinositol turnover leads to elevated intracellular phospholipid. This in turn could induce post-translational modification of the monomer subunits of E-NTPase/E-NTPDase in the intracellular milieu. Subsequently, the monomers are translocated to the membrane, depending on the tissue type(s) and functional requirement(s).

**Figure 2:**
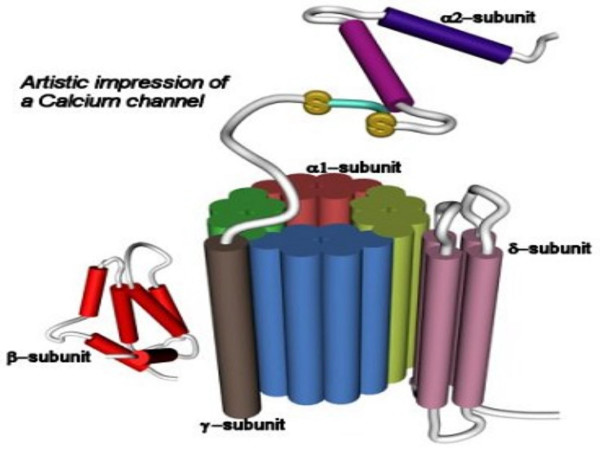
**Phase I: Activation. **Proposed model for E-NTPase/E-NTPDase in a functional state after oligomerization and assembly in the membrane, functioning as a gated channel.

**Figure 3:**
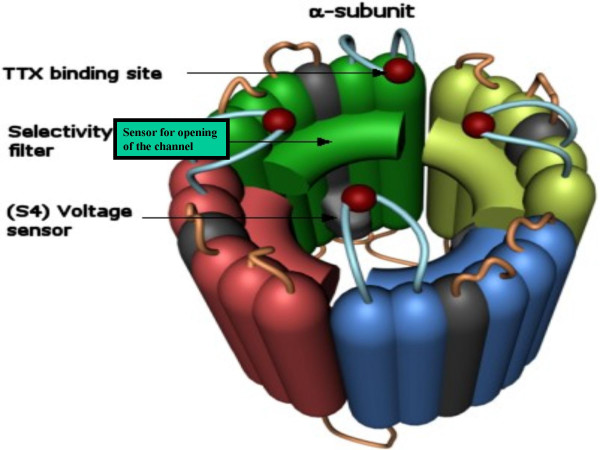
**Phase I: Activation. **The oligomerized E-NTPase/E-NTPDase would probably possess hypothetical sensors acting to open/close the gates.

**Figure 4:**
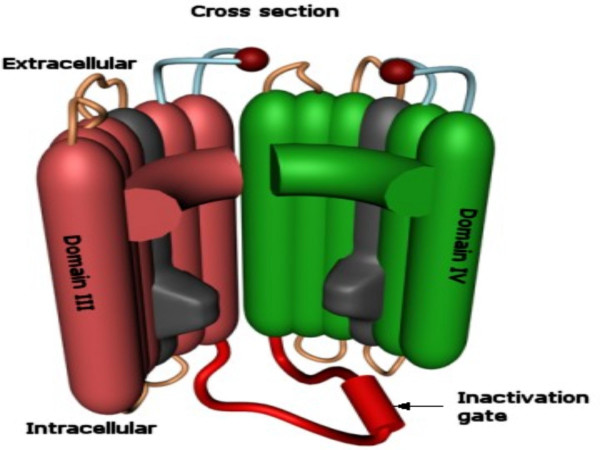
**Phase I: Activation. **Interior view of E-NTPase/E-NTPDase in a functional state in the membrane.

Membrane depolarization could locally alter protein conformation. This in turn could potentially induce post-translational modification in the (intracellular) monomer subunits of the E-NTPase/E-NTPDase, followed by translocation to the membrane (depending on the tissue type(s) and functional requirement(s)) (Fig. 1). Fig. 2 shows the proposed functional state of the E-NTPase/E-NTPDase after oligomerization and assembly in the membrane to form a gated Ca^2+^/Mg^2+ ^channel. Fig. 3, indicates that the oligomerized E-NTPase/E-NTPDase is likely to possess sensors to control the opening and closing of the Ca^2+^/Mg^2+ ^channel gate. Fig. 4, represents an interior view of the E-NTPase/E-NTPDase in the functional state after oligomerization and assembly in the membrane.

Probable energy sources and other significant factors are as follows. The source of extracellular nucleotides could be spontaneous release from dead cells or exocytosis from live/damaged cells [20]. In ocular ciliary epithelial cells, ATP is released in hypotonic conditions, and this release is inhibited by **NPPB **(5-nitro-2-(3-phenyl propylamine benzoic acid), a potent inhibitor of CFTR (cystic fibrosis transmembrane receptor) and p-glycoprotein mediated ATP release [21]. On the other hand, the endogenous CD39 of oocytes transforms under hypertonic conditions to a conformation mediating ATP transport to the extracellular environment, either by exocytosis or by acting as an ion channel [22,23]. However, under what conditions (hyper-or hypotonic) might CD39 assume an extracellular nucleotide hydrolyzing activity; and under those conditions, can this property be coupled to ion influx? This question remains unanswered.

At normal physiological temperature in presence of divalent succinyl CoA, Con A mediates the oligomerization of E-NTPase monomers/dimers to form a holoenzyme with enhanced activity. Eosin iodoacetamide (EIAA), a fluorescein iodoacetamide that forms thioester bonds with cysteine at neutral pH, enhances chicken gizzard ecto-ATPase activity [24].

There are ten conserved cysteine residues in E-NTPase (with additional cysteine residues in the N-terminal region that are known to mediate disulfide bond formation, essential in oligomerization). CD39, an ecto-Ca^2+^/Mg^2+ ^apyrase that hydrolyses ATP and ADP [25], forms tetramers and might act as a bivalent cation channel. However, the precise mechanism and functional properties are not known at present. CD39 expression is associated with ATP release; it was speculated that ATP release (along with drugs) into the extracellular milieu is followed by the hydrolysis of the extracellular nucleotides by CD39 [26].

Furthermore, native CD39 (ecto-ATP/Dase/ apyrase) forms tetramers upon oligomerization. Loss of either of the two transmembrane domains of rat CD39 ecto-ATP/Dase impairs enzyme activity. It has been suggested that the functional (holoenzyme) E-NTPase/E-NTPDase is a homotrimer in mammals.

Differences in enzyme activity among different species have been attributed to variations in the interaction among the monomers resulting in homotrimeric holoenzyme formation (66 kDa-ATPase) [27]. It seems clear that changes in the conformation of the E-NTPase/E-NTPDase could mediate changes in the channel transport function.

### Phase II: Ca^2+^/Mg^2+ ^Transport

**Figure 5:**
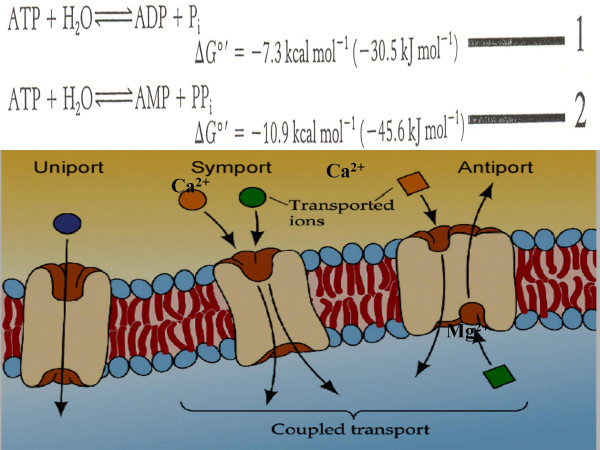
**Phase II: Ca^2+^/Mg^2+ ^Transport. **(A) Free energy released from ATP hydrolysis by E-NTPase on the outer membrane surface would yield -7.3 kcal mol^-1 ^**or **by formation of AMP by E-NTPDase would yield -10.9 kcal mol^-1^. (B) The energy is utilized for opening the channel formed by the E-NTPase/E-NTPDase, by altering the conformation of the sensors. This altered conformation has an inherent channel-opening effect; loss of the energy source causes the sensors to revert to the resting state, which corresponds to channel closing.

**Figure 6:**
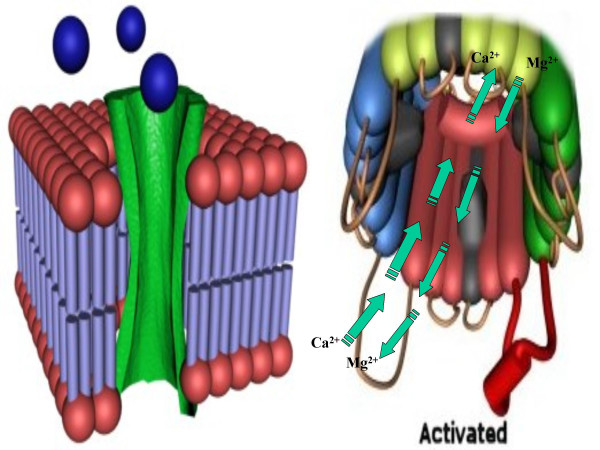
**Phase II: Ca^2+^/Mg^2+ ^Transport. **(A) Three-dimensional impression of the E-NTPase/E-NTPDase in vivo. (B) It is possible that Ca^2+ ^can enter the cell and excess Mg^2+ ^can leave via the influx/efflux mechanisms depicted in the figure.

Fig. 5a, illustrates the possible utilization of the energy released from [NTP] o /[NDP] o hydrolysis (-7.3 kcal mol^-1 ^or by formation of AMP, -10.9 kcal/mol^-1^) for opening the channel formed by the homo-oligomeric E-NTPase/E-NTPDase. This channel is postulated to open and close in response to energy availability (Fig. 5b). Fig. 6A, is an artist's impression of the three-dimensional configuration of the E-NTPase/E-NTPDase in vivo. Ca^2+ ^might enter the cell and excess Mg^2+ ^might leave by the influx and efflux mechanisms depicted in Fig 6b.

The opening of the slow inward Ca^2+ ^current channel in cardiac sarcolemma during the plateau phase of the action potential requires ATP [28]. Furthermore, protein kinase-A (PKA) dependent phosphorylation appears to mediate the increase in Ca^2+ ^influx in hormonal modulation of that process [29]. A similar model has been proposed for sodium channels in nerve membranes, in which a cycle of phosphorylation and dephosphorylation is proposed for opening and closing [30].

Other corroborating evidence implicating E-NTPase in Ca2+/Mg2+ transport via the gated channel is briefly summarised. Rat cardiac sarcolemmal E-NTPase has considerable sequence homology with the human platelet thrombospondin receptor CD36 [31]. An antibody directed against the purified E-NTPase blocked the increase in intracellular calcium concentration, implying that the E-NTPase plays an unknown but significant role in the delayed Ca^2+ ^influx or Mg^2+ ^efflux during the plateau phase of the action potential (Unpublished observation). Activation of E-NTPase by millimolar concentrations of Ca^2+ ^and electrical stimulation is linearly related to the contractile force developed in the myocardium [32]. Gramicidin S inhibits the E-NTPase activity and it attenuates the slow channel efflux in perfused frog left ventricles.

Based on these observations, we propose that E-NTPase might be involved in providing energy for Ca^2+^/Mg^2+ ^influx-efflux in the cardiac sarcolemma, opening the channel formed by the E-NTPase/E-NTPDase protein by altering the conformation of the sensors. The altered channel sensor conformation opens the channel; loss of the energy source allows the sensors to revert to the resting state, which corresponds to channel closing.

There are at least two Mg^2+ ^transport systems: (a) rapid transport down the concentration gradient and (b) efflux in low Ca^2+ ^Ringer during ventricular perfusion in vitro. In rat liver mitochondria, 50 nM cAMP or 250 μM ADP induced rapid loss of 6 mmol of Mg^2+^/mg protein coupled with the stimulation of ATP efflux. This effect was specific and was blocked by adenosine nucleotide translocase inhibitors. Evidently cAMP acts as a mobilizer of Mg^2+ ^in isolated rat liver mitochondria. Adenine nucleotide translocase is the cAMP target [33].

Myocardial Mg^2+ ^content is maintained at physiological level by the sarcolemmal transport system, which pumps Mg^2+ ^across the plasma membrane when the extracellular [Mg^2+^]_o _concentration is <1 mM and restores [Mg^2+^]_i _when the heart is perfused with Ringer buffer containing 5 × 10^-7 ^M Mg^2+^. Failure of either of these two transport mechanisms may result in a rise in [Mg^2+^]_i_, impairing the contractile machinery of the myocardium [34].

Gramicidin S inhibits total Mg^2+ ^efflux in the myocardium, while epinephrine restores Mg^2+ ^efflux and contractile force development in the frog ventricle perfused with 10 mM Mg^2+^. It should be pointed out that both E-NTPase activity and myocardial contraction and relaxation are inhibited by gramicidin S [35].

In the light of the evidence surveyed here, there would appear to be a significant functional role for activated E-NTPase in Ca^2+ ^influx and Mg^2+ ^efflux (or vice versa) in the myocardium.

### Phase III: Termination of the transport process

**Figure 7:**
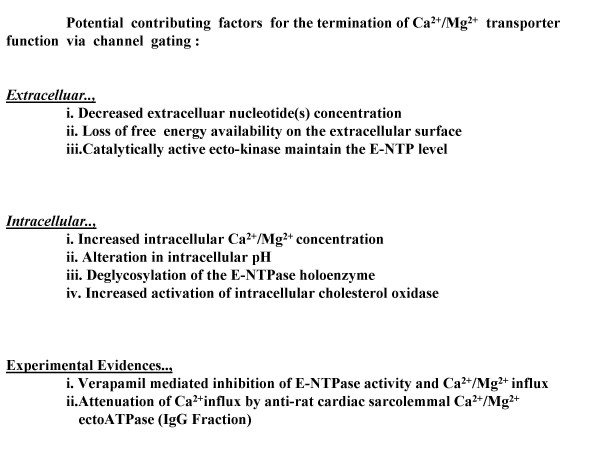
**Phase III: Termination of the transport processes. **(A) Several factors might contribute to the termination of Ca^2+^/Mg^2+ ^transport via channel gating by E-NTPase/E-NTPDase: extracelluar and Intracellular. Additional experimental evidence is mentioned. Decreased flow of Ca2+/Mg2+ due to closing of the channel gate.

Fig. 7 summarizes the possible means by which the transport process is terminated. There are several potential contributing factors that can be grouped into two categories, extracelluar and intracellular. Additional experimental evidence is indicated. Based on the heterologous expression of ecto-apyrase in COS cells in the presence of tunicamycin, glycosylation might be required for homo-oligomerization and nuclotidase activity. Conversely, deglycosylation might impair the E-type nucleotidase activity by weakening the monomer-monomer interaction and altering the tertiary and quaternary structures, result in the loss of holoenzyme. Essentially, glycosylation and deglycosylation of the ecto apyrase (HB6) monomer and the consequences for homodimer formation have been regarded as an on-off switch for ecto nucleotidase activity [36].

**Figure 8:**
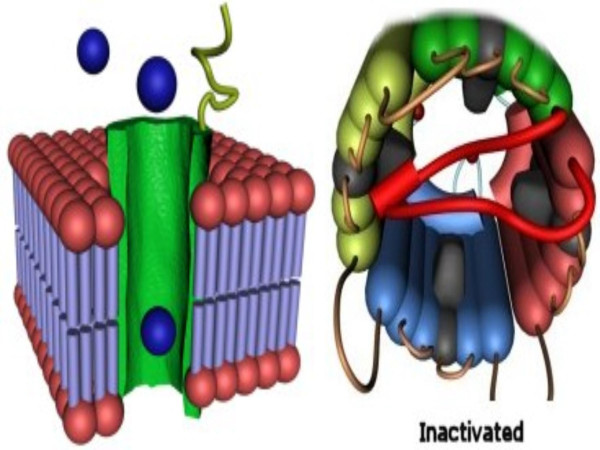
**Phase III: Termination of the transport processes. **(A) Three-dimensional impression of the E-NTPase/E-NTPDase in vivo when termination of the ion transport function commences. (B) Biochemical modifications of the E-NTPase/E-NTPDase oligomers such as deglycosylation would probably cause instability, leading to dissociation of the homo-oligomers. Disassembly of the functional molecule would ensue, closing the Ca^2+ ^influx and Mg^2+ ^efflux processes, as portrayed in the figure.

Fig. 8a is a three-dimensional impression of the ecto-ATPase in vivo at the termination of ion transport. Fig. 8b illustrates how biochemical modifications such as deglycosylation of the E-NTPase/E-NTPDase oligomers might cause dissociation of the homo-oligomers to individual monomers This is a potential mechanism for the disassembly of the functional channel and closure of Ca^2+ ^influx and Mg^2+ ^efflux. Also, an increase in membrane fluidity induced by cholesterol oxidation might cause defective association or disassociation due to weak interaction among the E-NTPase monomers, whereas increased membrane cholesterol might sustain higher E-NTPase activity. Oligomerization of E-NTPase and associated increase of activity could also be responsible for the rapid termination of the purinergic response mediated by extracellular ATP [37].

The extracellular nucleotide mediated activation of channel gating could be terminated by ecto (extracellular)-adenylate kinase, which catalyzes trans-phosphorylase activity (ADP+ADP→ ATP+AMP). This enzyme has a higher affinity for extracellular nucleotides than the dephosphorylating enzyme (E-NTPase/E-NTPDase) or ecto-nucleotide pyrophosphatase/phospho-diesterase (ATP→ AMP +ppi) [38].

As the transport process winds down, ecto-adenylate kinase mediated ATP generation might maintain the extracellular nucleotide level. However, the precise biochemical kinetic process by which this process is completed remains to be elucidated [39].

### Pathophysiological Significance of E-type nucleotidase mediated Ca^2+^/Mg^2+ ^transport

Impairment of E-Type nucleotidases during Ca^2+ ^paradox in isolated rat heart model warrants investigation of the molecular mechanism(s) involved. Knowledge obtained from these studies will elucidate the observed protective effects of anti-rat cardiac Ca^2+^/Mg^2+^-ecto-ATPase antibodies in ischemia reperfusion induced damage, which is a corollary of organ transplantation. Furthermore, the antiproliferative effect(s) of these antibodies in left anterior descending coronary artery smooth muscle cell(s) emphasize the need to explore more fully the hypothesis proposed in this article.

## Authors' contributions

HMS participated and provided the hypothetical scheme of the gating mechanism with appropriate literature. SK conceived and carried out experimental part of the investigation and formulating the hypotheses.

## Abbreviations

**E-NTPase **= Ecto or Extracellular Nucleotide triphosphatase; **E-NTPDase **= Ecto or Extracellular Nucleotide triphosphate diphosphohydrolase; **[Ca^2+^]_i _**= Intracellular Ca^2+^; **[NTP]_0 _**= Extracellular Nucleotide triphosphate; **[NDP]**_0 _= Extracellular Nucleotide diphosphate; **ROCC **= Receptor Operated Ca^2+ ^channel; **SER **= Sarco (Endo) plasmic reticulum; **[cAMP]_i _**= Cytoplasmic or intracellular cAMP; **PMCA **= Plasma membrane Ca^2+^pump ATPase.**VOCC **= voltage-operated Ca^2+ ^channel; **LGCCS **= Ligand gated non-specific cation channels. **RACC = **Stretch/Receptor activated non-specific Ca^2+^channels. **SUR **= Sulfonylurea Receptor Proteins. **CD36 **= Thrombospondin receptor on platelets. **CD39 **= Ecto Ca^2+^/Mg^2+^apyrase.
